# Supplementary figures and images for: Conserved Induction of Distinct Antiviral Signalling Kinetics by Primate Interferon Lambda 4 Proteins
Source: Front Immunol. 2021 Nov 18;12:772588. doi: 10.3389/fimmu.2021.772588 (PMC8636442; doi:10.3389/fimmu.2021.772588)

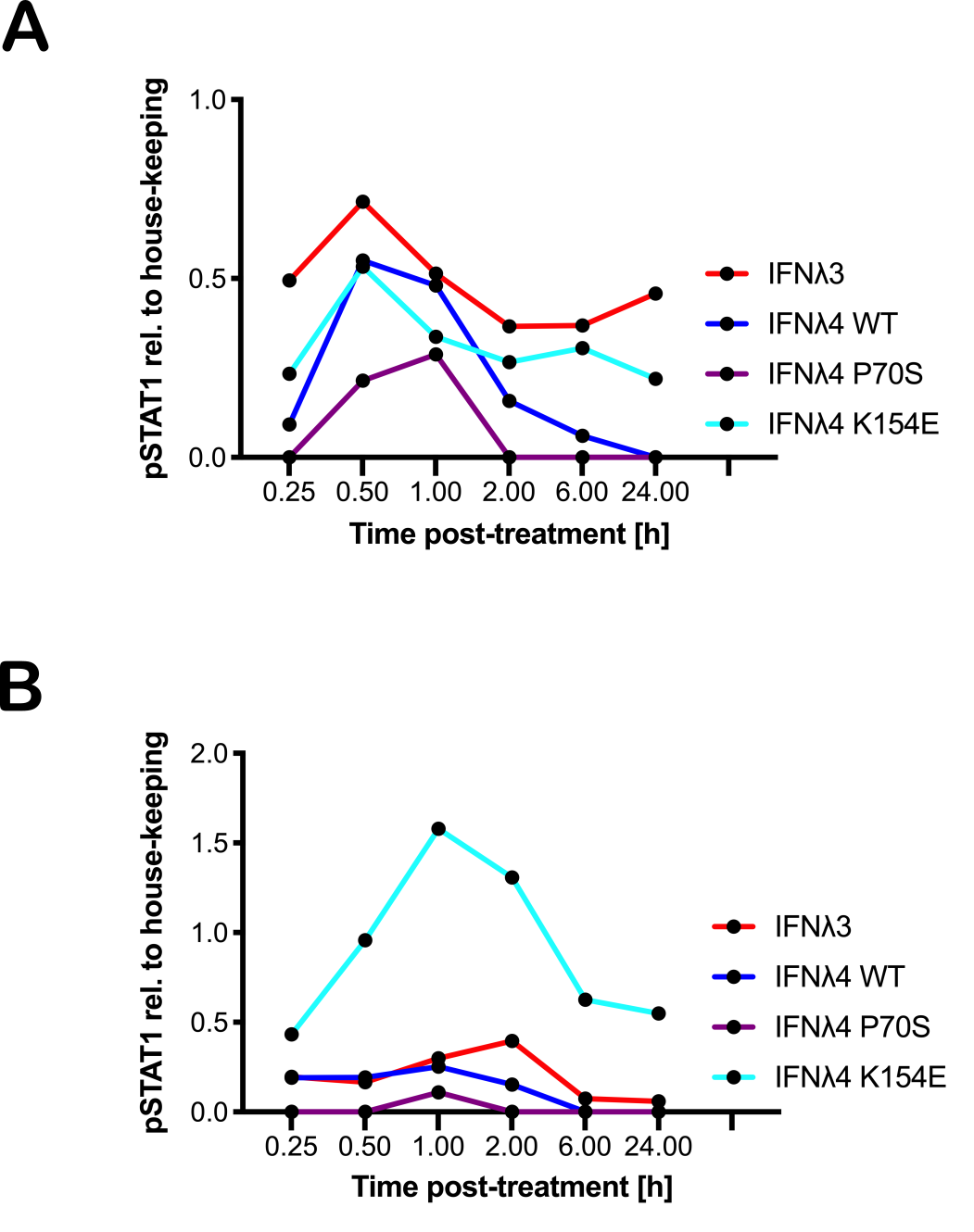

Supplement: Supplementary Figure 1 — pSTAT1 quantification over time for IFNλs on liver and gut cells. Quantification of pSTAT1 from images in Figure 1 compared to house-keeping control and background levels was carried out by densitometry analysis for HepaRG (A) and T84 (B) cells (IFNλ3-HiBiT [red], IFNλ4-HiBiT: WT [blue], P70S [purple], and K154E [cyan]. [file Image_1.tiff]

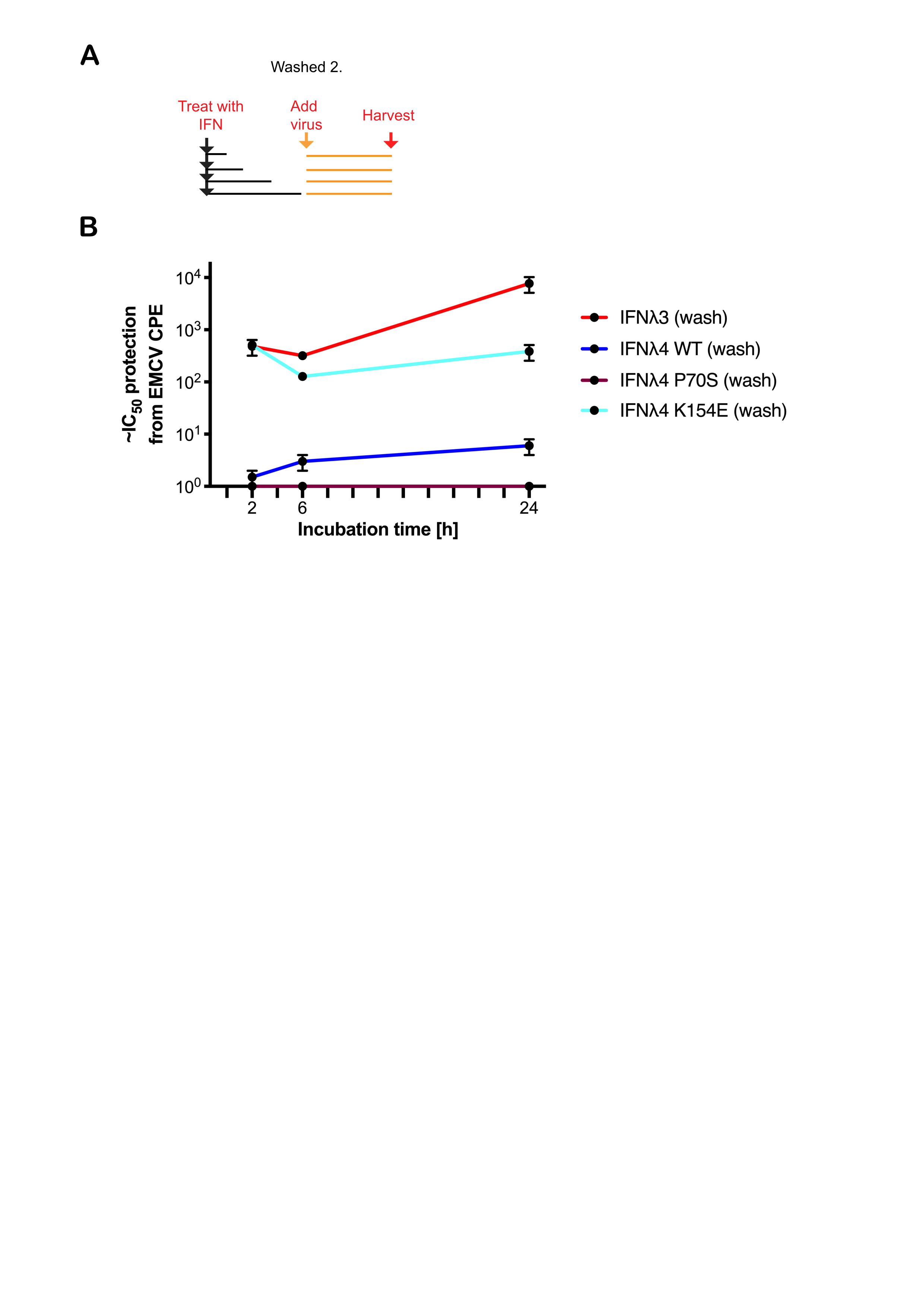

Supplement: Supplementary Figure 2 — Effect of IFN incubation time on kinetics of human IFNλ variants. HepaRG cells were stimulated with IFNλs: IFNλ3-HiBiT (red), IFNλ4-HiBiT variants: WT (blue), P70S (purple), and K154E (cyan) at indicated times (2, 6 or 24h) before supernatant was removed and rinsed with PBS before being replaced with fresh media not containing virus (A). Stimulated cells were incubated until 24h after IFNλ incubation prior to infection with EMCV and antiviral activity was read 24hpi (B). Error bars represent the mean ± SEM from 2 biological replicates. [file Image_2.tiff]
